# Supplementary material for: Policies for healthy ageing in response to climate change: Protocol of a systematic review
Source: PLoS One. 2025 Apr 30;20(4):e0323069. doi: 10.1371/journal.pone.0323069 (PMC12043174; doi:10.1371/journal.pone.0323069)
Supplement: S2 Appendix 2 — (DOCX) [file pone.0323069.s002.docx]

**Appendix 2. Search strings and strategy generated across key search databases**

Search on EBSCOhost

| String name/number | String |
| --- | --- |
| S1 | healthy ageing or healthy aging or aging well or ageing well or older adults or elderly or aging population or successful aging or aged individuals or senior citizens or older people or late-life adults |
| S2 | climate change or global warming or greenhouse effect or climatic changes or environmental change or extreme weathers |
| S3 | policy or policies or law or laws or legislation or climate change adaptation or governance or intervention or strategy or climate mitigation or emergency preparedness or disaster risk reduction |
| Final | S1 AND S2 AND S3 |

Search on MEDLINE/PubMed

| String name/number | String |
| --- | --- |
| S1 | healthy ageing or healthy aging or aging well or ageing well or older adults or elderly or aging population or successful aging or aged individuals or senior citizens or older people or late-life adults |
| S2 | climate change or global warming or greenhouse effect or climatic changes or environmental change or extreme weathers |
| S3 | policy or policies or law or laws or legislation or climate change adaptation or governance or intervention or strategy or climate mitigation or emergency preparedness or disaster risk reduction |
| Final | ((healthy ageing or healthy aging or aging well or ageing well or older adults or elderly or aging population or successful aging or aged individuals or senior citizens or older people or late-life adults) AND (climate change or global warming or greenhouse effect or climatic changes or environmental change or extreme weathers)) AND (policy or policies or law or laws or legislation or climate change adaptation or governance or intervention or strategy or climate mitigation or emergency preparedness or disaster risk reduction) |

Search on Web of Science

| String name/number | String |
| --- | --- |
| S1 | healthy ageing or healthy aging or aging well or ageing well or older adults or elderly or aging population or successful aging or aged individuals or senior citizens or older people or late-life adults |
| S2 | climate change or global warming or greenhouse effect or climatic changes or environmental change or extreme weathers |
| S3 | policy or policies or law or laws or legislation or climate change adaptation or governance or intervention or strategy or climate mitigation or emergency preparedness or disaster risk reduction |
| Final | ((ALL=(healthy ageing or healthy aging or aging well or ageing well or older adults or elderly or aging population or successful aging or aged individuals or senior citizens or older people or late-life adults)) AND ALL=( climate change or global warming or greenhouse effect or climatic changes or environmental change or extreme weathers)) AND ALL=( policy or policies or law or laws or legislation or climate change adaptation or governance or intervention or strategy or climate mitigation or emergency preparedness or disaster risk reduction) |

Search on SCOPUS

| String name/number | String |
| --- | --- |
| S1 | healthy ageing or healthy aging or aging well or ageing well or older adults or elderly or aging population or successful aging or aged individuals or senior citizens or older people or late-life adults |
| S2 | climate change or global warming or greenhouse effect or climatic changes or environmental change or extreme weathers |
| S3 | policy or policies or law or laws or legislation or climate change adaptation or governance or intervention or strategy or climate mitigation or emergency preparedness or disaster risk reduction |
| Final | ((healthy ageing or healthy aging or aging well or ageing well or older adults or elderly or aging population or successful aging or aged individuals or senior citizens or older people or late-life adults) AND (climate change or global warming or greenhouse effect or climatic changes or environmental change or extreme weathers)) AND (policy or policies or law or laws or legislation or climate change adaptation or governance or intervention or strategy or climate mitigation or emergency preparedness or disaster risk reduction) |
