# Supplementary material for: Policies for healthy ageing in response to climate change: Protocol of a systematic review
Source: PLoS One. 2025 Apr 30;20(4):e0323069. doi: 10.1371/journal.pone.0323069 (PMC12043174; doi:10.1371/journal.pone.0323069)
Supplement: S2 Appendix 3 — (DOCX) [file pone.0323069.s003.docx]

Appendix 4. Data extraction sheet

| ID | Bibliometric information | | | | | | | | Primary results | | Other information | | | |
| --- | --- | --- | --- | --- | --- | --- | --- | --- | --- | --- | --- | --- | --- | --- |
|  | Author(s) | Year* | Journal | Country** | Study setting | Design | Sample size | Population | Objective 1 | Objective 2 | Year*** | Purpose of policy | Target population | Type of policy (i.e., whether adopted or recommended) |
| 1 |  |  |  |  |  |  |  |  |  |  |  |  |  |  |
| 2 |  |  |  |  |  |  |  |  |  |  |  |  |  |  |
| 3 |  |  |  |  |  |  |  |  |  |  |  |  |  |  |
| 4 |  |  |  |  |  |  |  |  |  |  |  |  |  |  |
| 5 |  |  |  |  |  |  |  |  |  |  |  |  |  |  |
| 6 |  |  |  |  |  |  |  |  |  |  |  |  |  |  |
| 7 |  |  |  |  |  |  |  |  |  |  |  |  |  |  |
| 8 |  |  |  |  |  |  |  |  |  |  |  |  |  |  |
| 9 |  |  |  |  |  |  |  |  |  |  |  |  |  |  |
| 10 |  |  |  |  |  |  |  |  |  |  |  |  |  |  |
| 11 |  |  |  |  |  |  |  |  |  |  |  |  |  |  |
| 12 |  |  |  |  |  |  |  |  |  |  |  |  |  |  |
| 13 |  |  |  |  |  |  |  |  |  |  |  |  |  |  |
| 14 |  |  |  |  |  |  |  |  |  |  |  |  |  |  |
| 15 |  |  |  |  |  |  |  |  |  |  |  |  |  |  |
| 16 |  |  |  |  |  |  |  |  |  |  |  |  |  |  |
| 17 |  |  |  |  |  |  |  |  |  |  |  |  |  |  |
| 18 |  |  |  |  |  |  |  |  |  |  |  |  |  |  |

*Year of study’s publication; **country or countries where the study was undertaken; ***Year in which the policy was recommended or took effect
