# Supplementary material for: Policies for healthy ageing in response to climate change: Protocol of a systematic review
Source: PLoS One. 2025 Apr 30;20(4):e0323069. doi: 10.1371/journal.pone.0323069 (PMC12043174; doi:10.1371/journal.pone.0323069)
Supplement: Attachment — Submitted filename: Response to Reviewers.doc [file pone.0323069.s005.doc]

**Response to Reviewers**

**General response to editor and reviewers**

We are pleased to see the expert feedback provided by the editor and reviewers; we appreciate the time spent on our work. All the suggestions are constructive and plausible, and the comments from both reviewers are very specific. We are happy to revise the work as suggested. Our responses are highlighted in yellow as follows.

**Reviewer #1:** Well done on your Protocol of a Systematic Review. Please find below specific comments to address.

INTRODUCTION

1. Reference needed after first sentence

**Response** – thank you; a relevant reference has been inserted at the end of the sentence.

2. Reference needed second paragraph, third sentence

**Response** – A relevant reference has been inserted at the end of the sentence.

3. Insert "globally" to third paragraph sentence- Because of population ageing, the population of older adults aged 65 years or older "globablly" is expected to more than double by 2050.

**Response** – this is helpful. We’ve inserted “globally” in that sentence.

4. Reference needed third paragraph, third sentence

**Response** – A relevant reference has been inserted at the end of the sentence.

5. Remove paragraph 4 related to people of all ages as unclear why this is needed?

**Response** – We agree with you. We’ve removed the sentences.

6. Remove from healthy ageing paragraph sentence "Hence, the ideal policies for healthy ageing in response to climate change should be targeted at every ageing individual"

**Response** – It’s removed. Thank you.

7. Include at end of healthy ageing paragraph "Research in the past decades shows increased advocacy for healthy ageing policies in response to climate change [14, 15]. While some studies report policies already developed for fostering healthy ageing in contexts experiencing climate change events, others have reported policies recommended by experts for the same purpose [14, 16]."

**Response** – We have moved the quoted text to the end of the healthy ageing paragraph.

METHODS

8. Correct spelling "Meta-Analyses" in (Review protocol and registration)

**Response** – thanks for seeing this error. It’s corrected.

9. Include "also" in (Types of studies)- Although systematic reviews may focus on peer-reviewed articles, we plan to "also" include grey literature

**Response** – thank you. It’s corrected

10. Provide exclusion criteria for (Types of studies)

**Response** – we’ve provided exclusion criteria at the end of the paragraph

11. Remove "global warming" as this is not mentioned in introduction

**Response** – we’ve removed it.

12. Unclear if the current search strategy will identify (Types of comparators)- barriers to adoption healthy ageing, please consider describing how this will be achieved

**Response** – We’ve clarified the comparators in the paragraph.

13. Include "people with a disability" (Types of outcome measures)- brackets in final sentence

**Response** – that was helpful.

14. (First stage screening)- Study participants= Remove "often people aged 18 years or older"

**Response** – I like the specificity of your comments; you’re an excellent reviewer. Thank you.

15. Include detail information about literature uses 50 years as the minimum old age in Introduction, as currently you talk about older adults as 65 years or older

**Response** – Thank you. We’ve revised the section appropriately.

16. Study Participants= Remove "will be potentially eligible. Such materials"

**Response** – thank you. It’s done

17. (Risk of Bias Assessment)= Ensure you write in full before using abbreviations e.g. Critical Appraisal Skills Programme (CASP) or Joanna Briggs Institute (JBI)

**Response** – thank you. We’ve corrected the error

18. Include "either" to first sentence Risk of Bias Assessment- The risk of bias and quality of studies will be assessed with "either" the

**Response** – we agree with you. We’ve made the change

DISCUSSION AND CONCLUSION

19. Keep consistent terminology throughout paper, change programmes in first sentence to "policies"

**Response** – thank you. We’ve made the change

20. Unclear who the specific stakeholders are? (e.g. general population/individuals, parliament, organisations, academics etc)

**Response** – we’ve named the stakeholders in parentheses

21. Reword final sentence suggestion "Finally, this review will highlight areas where future research and policy dialogues are needed to foster successful implementation of healthy ageing policies."

**Response** – we’ve revised the sentence to make it more concise.

**Reviewer #2: 1.** The introduction provides a partly valid rationale for the proposed study, clearly justifying research questions; however, some parts need more clarity:

- Some broad statements should be backed by specific examples/evidence. For example, "Research in the past decades shows increased advocacy for healthy ageing policies in response to climate change [14, 15]" should be more specific such as what kind of policies or frameworks have been developed and in which contexts (e.g., WHO frameworks, national-level policies).

**Response** – We agree with you. We’ve identified two frameworks in the following paragraphs.

- The phrase “climate change accompanies” is inappropriate. Climate change increases the frequency and intensity of extreme weather events rather than simply accompanying them.

**Response** – thank you. We’ve revised the sentence

- The pathways through which climate change affects health are not well defined. "It threatens biodiversity, increases the risk of mortality, and makes communities more vulnerable to respiratory diseases." The phrase “makes communities more vulnerable” is too broad and lacks specific mechanisms. Data should support the statement.

**Response** – we’ve revised the sentence and provided references for each specific pathway.

2. The search strategy is well-structured, but some important keywords are missing in the search strings.

In your appendix 2 "Search strings and strategy generated across key search databases" consider including the following terms:

- S1 (population): Add: older adults, elderly, aging population, successful aging, aged individuals, senior citizens, older people, late-life adults

- S2 (climate): environmental change, extreme weathers (may be specific to storms, floods, heatwaves, heat stress, rising temperatures, extreme heat, cyclones, disasters, cold waves, extreme cold, winter storms, urban heat islands, rising sea levels)

- S3 (policy): climate change adaptation, governance, intervention, strategy, climate mitigation, emergency preparedness, disaster risk reduction

**Response** – thank you. We’ve updated the search terms and strategies as suggested. Please see Appendix 2.
